# Ruptured Baker’s Cyst Demystified: Current Evidence, Diagnostic Strategies, and Treatment Options for an Under-Recognized Condition

**DOI:** 10.7759/cureus.101686

**Published:** 2026-01-16

**Authors:** Hassan Zmerly, Luigi Di Lorenzo, Vanessa Mahfouz, Fabio Valerio Sciarretta, Francesco Pegreffi

**Affiliations:** 1 Department of Orthopedics, Villa Erbosa - Gruppo San Donato, Bologna, ITA; 2 Faculty of Medicine and Surgery, Link Campus University, Rome, ITA; 3 Department of Orthopedics, San Pier Damiano Hospital, GVM Care and Research, Faenza, ITA; 4 Department of Life Sciences, Health, and Health Professions, Link Campus University, Rome, ITA; 5 Department of Orthopedics, Azienda Ospedaliera di Modena, Modena, ITA; 6 Department of Orthopedics, Clinica Nostra Signora della Mercede, Rome, ITA; 7 Department of Medicine and Surgery, Kore University of Enna, Enna, ITA; 8 Recovery and Functional Rehabilitation Unit, Umberto I Hospital, Enna, ITA

**Keywords:** baker's cyst, deep vein thrombosis (dvt), popliteal cyst, popliteal mass, ruptured baker's cyst

## Abstract

Rupture of a Baker’s cyst is an under-recognized condition that may closely mimic deep vein thrombosis, often leading to diagnostic uncertainty and delayed management. Although popliteal cysts are common findings associated with degenerative or inflammatory knee disorders, their rupture represents an uncommon but clinically significant complication. Ruptured cysts typically present with acute calf swelling and pain due to extravasation of synovial fluid into the intermuscular planes. The condition may mimic venous thrombosis (pseudo-thrombophlebitis), making diagnostic accuracy essential. Duplex ultrasonography is the first-line modality, primarily to exclude deep vein thrombosis, while musculoskeletal ultrasound and MRI help identify fluid dissection and residual cyst anatomy. Most cases are self-limiting and respond well to conservative treatment, including rest, ice, compression, elevation, and anti-inflammatory medication. Image-guided aspiration, corticosteroid injection, and ultrasound-guided fenestration may be considered in persistent or recurrent cases. Surgical management, most commonly arthroscopic treatment of intra-articular pathology with cyst decompression, is reserved for refractory symptoms or when the underlying joint lesion requires correction. Rare complications, such as compartment syndrome, necessitate prompt recognition and intervention. This review aims to synthesize current evidence on the clinical presentation, diagnostic challenges, imaging strategies, and management options for ruptured Baker’s cysts.

## Introduction and background

Baker’s cysts, or popliteal fossa cysts, are very common lesions of the knee. They occur predominantly in individuals aged 35 to 70 years, with prevalence increasing progressively with age as a consequence of degenerative joint changes and declining capsular integrity [1-3].

**Figure 1:**
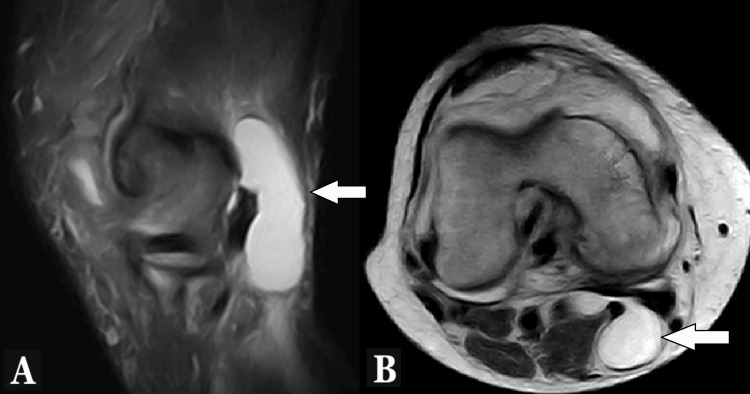
MRI of a Baker’s cyst. MRI shows synovial-fluid distension of the gastrocnemius-semimembranosus bursa, typically maintaining a communication with the knee joint via a posterior capsular opening on the sagittal view (A) and the axial view (B).

A popliteal cyst is defined as a synovial-fluid distension of the gastrocnemius-semimembranosus bursa, typically maintaining a communication with the knee joint via a posterior capsular opening [4-6]. The diagnosis is based on clinical presentation, ultrasound, and MRI examination (Figure 1). Management is generally conservative [7].

These cysts are strongly associated with underlying intra-articular pathologies, including osteoarthritis, rheumatoid arthritis, and other structural abnormalities. Up to 94% of Baker’s cysts in adults are linked to intra-articular degenerative changes (e.g., meniscal tears, cartilage injuries, and anterior cruciate ligament lesions) [6]. Cyst enlargement may occur in the setting of persistent joint effusion, mechanical irritation, or recurrent microtrauma. Iatrogenic factors, such as post-infiltrative or post-operative fluid accumulation, have also been reported [5-7].

Rupture of a Baker’s cyst is an uncommon event but carries clinical relevance. Predisposing factors include abrupt increases in intracystic pressure resulting from rapid synovial fluid accumulation or vigorous physical activity [8-11]. When rupture occurs, the clinical presentation may be challenging, and delayed recognition can lead to complications such as compartment syndrome [12].

This review aims to provide an updated synthesis of the available literature on the assessment and management of ruptured Baker’s cysts, highlighting key clinical and imaging features to improve diagnostic accuracy and offering evidence-based recommendations to guide optimal treatment and clinical decision-making.

## Review

Methods

A focused search of the MEDLINE/PubMed database was conducted from 1 January 2000 to 31 October 2025, to ensure the inclusion of the most recent and relevant evidence. The search strategy employed a combination of the following keywords: popliteal cyst, Baker’s cyst, cyst rupture, knee, diagnosis, management, and treatment. A narrative review of the retrieved English-language literature was subsequently performed. Relevant evidence was synthesized and descriptively summarized.

Anatomy and pathophysiology

A popliteal cyst results from the accumulation of synovial fluid outside the knee joint, forming a fluid-filled distension between the semimembranosus tendon and the medial head of the gastrocnemius [13]. This occurs when increased intra-synovial pressure causes the joint capsule to bulge at a structurally weaker area in the posterior knee. Synovial fluid originating from degenerative or inflammatory knee diseases follows the path of least resistance and enters the gastrocnemius-semimembranosus bursa [13-16].

The cyst arises from this bursa, which maintains a characteristic communication with the knee joint through a posterior capsular opening. In adults with knee effusion, a functional one-way valve mechanism forms at this communication: synovial fluid is driven into the bursa under pressure but cannot easily return to the joint. The dynamic interaction between the semimembranosus and gastrocnemius tendons during knee motion contributes to this valve effect. Consequently, intra-articular pathologies, such as meniscal tears, cartilage lesions, or arthritis, can lead to progressive fluid accumulation within the bursa, promoting cyst formation and enlargement.

Although many popliteal cysts remain asymptomatic, several factors may predispose to rupture. A rapid increase in intra-cystic pressure caused by sudden effusion accumulation, strenuous physical activity, or direct trauma can exceed the tensile strength of the cyst wall [3,13-15]. Large cyst size and chronic inflammation, as seen in rheumatoid arthritis, may further increase susceptibility to rupture [16-18].

When rupture occurs, the cyst wall tears and synovial fluid extravasates into the adjacent calf compartments, frequently dissecting along intermuscular planes, such as between the gastrocnemius heads or within deep fascial layers [16-20]. The presence of synovial fluid in these tissues triggers a robust inflammatory reaction due to its bioactive mediators and proteolytic enzymes. Unlike a classic hematoma, the extravasated synovial fluid provokes marked inflammation, contributing to the dramatic clinical presentation.

In children, the pathophysiology differs slightly: cyst formation is believed to arise from herniation of the joint capsule rather than a valve mechanism, often occurring without an identifiable intra-articular lesion.

Overall, cyst development reflects a combination of increased intra-articular pressure, functional valve dynamics, and underlying joint pathology, whereas rupture results from abrupt pressure elevation or trauma, leading to fluid dissection through the calf tissues [18-21].

Clinical presentation

Rupture of a popliteal (Baker’s) cyst typically manifests with acute pain, swelling, and a sensation of tightness in the calf. These symptoms result from the abrupt extravasation of synovial fluid into the intermuscular planes of the leg [22-24]. Patients often describe a sudden onset of calf discomfort, occasionally accompanied by the sensation of fluid “running down” the leg. Walking may become difficult due to pain and tension within the calf musculature, and knee stiffness or discomfort may also be present.

One recognizable but uncommon clinical sign is the crescent sign: a gravity-dependent ecchymosis appearing below the medial malleolus, produced by the distal tracking of synovial fluid along fascial planes [25]. Although highly suggestive when present, it is not observed in all cases. A positive Homan’s sign, a clinical test used to detect the presence of deep vein thrombosis (DVT) and characterized by pain on forced ankle dorsiflexion, may also be elicited and can further contribute to diagnostic confusion with DVT [3,26].

Physical examination may reveal swelling or tenderness in the popliteal fossa if part of the cyst remains intact, although complete decompression following rupture may obscure palpable findings. Signs of underlying knee pathology, such as joint line tenderness or crepitus, may also be present.

Diagnosis

The diagnostic evaluation of a suspected ruptured Baker’s cyst focuses on confirming the rupture and excluding other conditions, particularly DVT, which shares similar clinical features [27-31]. Because clinical assessment alone is often insufficient, imaging plays a central role.

Ultrasonography

Duplex ultrasonography is the first-line investigation, primarily because it reliably excludes DVT by demonstrating normal venous compressibility and blood flow [1]. Once DVT is ruled out, musculoskeletal ultrasound can evaluate the popliteal region for evidence of a cyst or fluid extravasation.

Typical findings of an intact Baker’s cyst include an anechoic or hypoechoic fluid collection located between the semimembranosus tendon and the medial head of the gastrocnemius [1]. Following rupture, ultrasound may show fluid tracking inferiorly along the calf’s intermuscular planes even if the cyst has partially or completely decompressed [9]. In some cases, remnants of the cyst wall or a disrupted cyst margin may be visualized, although these features are not always present after rupture [9].

Because it is rapid, widely accessible, and non-invasive, ultrasonography remains the most practical initial imaging modality and provides essential information for both diagnosis and differential diagnosis.

Magnetic Resonance Imaging (MRI)

**Figure 2:**
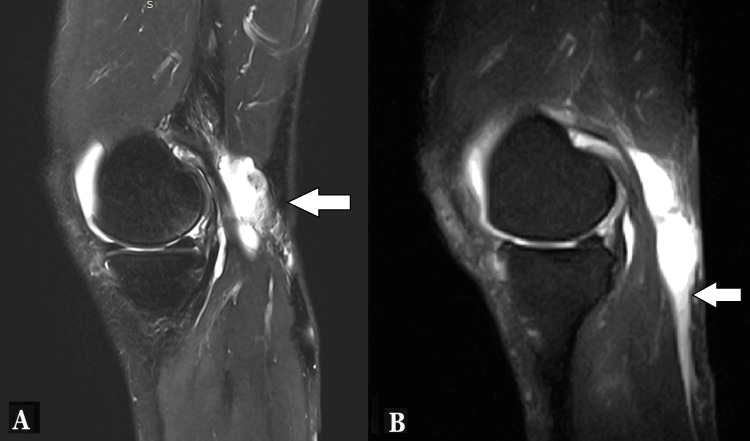
MRI of a ruptured Baker’s cyst. MRI sagittal images of a ruptured Baker’s cyst showing irregular fluid collection (A) with dissection along the fascial plane (B).

When ultrasonography yields equivocal findings or when a more detailed anatomical assessment is required, MRI serves as the gold standard. MRI can directly visualize the popliteal cyst, identify the rupture site, and delineate the pattern and extent of fluid dissection within the calf [1,4,9]. Ruptured cysts typically appear as complex fluid collections following fascial or intermuscular planes (Figure 2). MRI also provides excellent characterization of intra-articular abnormalities, such as meniscal tears, cartilage defects, and synovial proliferation, which contribute to cyst formation and persistence.

Recent MRI-based studies have identified features associated with complicated cysts, including increased cyst volume, marked joint effusion, medial meniscal extrusion, and synovial proliferation, the latter being the most significant predictor of complexity [21].

Plain Radiography

X-rays have a limited role in diagnosing ruptured cysts but may reveal associated degenerative changes, calcifications, or other bony abnormalities that support the clinical context of underlying knee pathology.

Other Imaging Modalities

Although less commonly employed, advanced imaging techniques such as radionuclide scans have been used in selected cases to detect fluid tracking when other modalities are inconclusive [31,32].

A practical diagnostic pathway begins with duplex ultrasonography to exclude DVT, followed, if needed, by musculoskeletal ultrasound or MRI for definitive visualization of the cyst and associated fluid extravasation. This systematic approach ensures diagnostic accuracy while avoiding unnecessary anticoagulation or delayed recognition of alternative pathologies.

Management

Management of ruptured popliteal (Baker’s) cysts is primarily conservative, as the condition is usually self-limiting and the extravasated synovial fluid is progressively reabsorbed over time [32].

The main therapeutic objectives are to alleviate pain, reduce inflammation, promote fluid resorption, and prevent complications. A structured approach is essential, particularly in cases where symptoms mimic DVT or when complications such as compartment syndrome may arise.

Conservative Management

Conservative treatment remains the cornerstone of therapy and is appropriate for the vast majority of patients [33]. Initial management focuses on symptom control through rest and activity modification to minimize calf pain, along with ice application during the acute inflammatory phase, compression bandaging to reduce local swelling, and leg elevation to facilitate fluid resorption [34]. Nonsteroidal anti-inflammatory drugs (NSAIDs) or other analgesics are commonly used to alleviate pain and inflammation. As the extravasated synovial fluid is progressively reabsorbed by surrounding tissues, symptoms generally improve within several weeks, with most patients achieving significant relief within four to 12 weeks [9]. Once acute symptoms diminish, gentle range-of-motion exercises and physiotherapy may be introduced to restore knee mobility and address biomechanical factors contributing to joint effusion. Reassuring patients is also crucial, as the sudden onset of pain and calf swelling may be alarming; clinicians should emphasize the benign and self-limiting nature of the condition and the expected trajectory of recovery.

Image-Guided Interventions

When symptoms persist despite conservative measures or when the cyst remains tense and symptomatic, image-guided procedures may be considered before proceeding to surgical options. Ultrasound-guided aspiration can provide temporary relief by decompressing the cyst cavity [3], and this is often followed by corticosteroid injection either into the cyst or intra-articular to reduce synovial inflammation and slow recurrent fluid accumulation. Although this approach may provide symptomatic improvement, recurrence is common in patients with underlying osteoarthritis or meniscal pathology, where persistent joint effusion drives cyst formation [3]. More recently, minimally invasive techniques combining ultrasound-guided cyst fenestration with corticosteroid injections have been described [10]. Fenestration involves needling and widening the valve-like communication between the cyst and the joint to promote continuous drainage, followed by a steroid injection to reduce inflammation. Early evidence suggests this combined approach may reduce recurrence compared with aspiration alone, though long-term outcomes remain limited. Novel approaches such as platelet-rich plasma (PRP) injections and doxycycline sclerotherapy have been reported in isolated cases; however, the evidence is preliminary and insufficient to support routine use.

Surgical Management

Surgical treatment is based on clinical manifestation, the response to conservative treatment, and the MRI characteristic including the shape, size, localization, and the internal structure of the cyst. The surgical treatment for a Baker’s cyst can be done by an arthroscopic or open approach; however, there is a lack of data in the literature regarding specific surgical procedures for a ruptured Baker’s cyst.

Arthroscopic Approach

Arthroscopic management is widely regarded as the preferred surgical option for patients who do not respond to conservative or minimally invasive treatments, particularly when an identifiable intra-articular lesion is responsible for persistent cyst formation [33-37]. The procedure is performed with the patient in a supine or floppy lateral position using a standard 30° scope and the three-approach antero-lateral, antero-medial, and postero-medial portal [26].

The antero-lateral portal allows the visualization of the communication between the joint and the Baker’s cyst, while the antero-medial and postero-medial are used for instruments (basket, shaver, and cautery) [26]. The scope can also be switched to the postero-medial portal and advanced to the posterior and distal aspect to better visualize the cyst wall [34]. The water pump pressure must not exceed 50 mmHg to avoid leakage through the ruptured cyst, and the calf must be continuously monitored [34]. Care must be taken to avoid neuro-vascular iatrogenic damage.

The arthroscopic approach offers the advantage of simultaneously addressing both the underlying joint pathology and the mechanism that contributes to cyst persistence. Intra-articular abnormalities, such as meniscal tears or cartilage defects, can be treated arthroscopically, while the communication between the joint space and the gastrocnemius-semimembranosus bursa can be enlarged or unroofed to eliminate the one-way valve mechanism that promotes cyst formation [10]. By improving fluid egress from the cyst back into the joint, this technique aims to prevent reaccumulation and reduce recurrence. The cystectomy can be performed by cautery of the cyst wall from the postero-medial portal [26].

Arthroscopic procedures generally yield favorable outcomes, although recurrence may still occur, particularly in patients with advanced osteoarthritis where persistent effusion remains a problem [26,34,37,38]. Treating the intra-articular pathology is therefore essential to optimize long-term results.

Open Surgical Excision

Open surgical excision is now performed far less frequently, as recurrence rates are significantly higher when the underlying intra-articular pathology is not addressed. Nevertheless, it may still be considered in specific circumstances, such as isolated cysts without associated joint disease, select pediatric cases, or situations in which arthroscopy is contraindicated. The procedure involves direct removal of the cyst through a posterior approach [35]; however, it carries notable risks, including potential neurovascular injury due to the anatomical complexity of the popliteal fossa, postoperative stiffness, and recurrence. These limitations have led to a progressive decline in its use, with open excision reserved for highly selected cases in which arthroscopic management is not feasible or appropriate [26].

Discussion

Although the rupture of a popliteal (Baker’s) cyst may present with acute and sometimes dramatic symptoms, the overall prognosis is generally favorable. In most patients, the inflammatory response induced by the extravasated synovial fluid gradually subsides as the fluid is reabsorbed, and complete resolution of symptoms is typically achieved with conservative treatment alone [1].

Popliteal cysts have been found in 4.7% to 37% of adults with asymptomatic knees [32]. Ruptured Baker’s cyst is rare; however, in a study of 680 patients with posterior knee pain evaluated by ultrasound, Charnock et al. [39] identified Baker’s cysts in 342 cases, while cyst rupture was observed in five cases.

Complications from ruptured Baker’s cysts are very rare but may be clinically significant. Large unruptured cysts may cause compressive symptoms, including venous obstruction or neural irritation. In rare cases, a ruptured cyst may result in compartment syndrome of the calf, a serious condition caused by extensive synovial fluid extravasation leading to elevated intra-compartmental pressure. Reported manifestations include severe pain, tense swelling, neurovascular impairment, and foot drop, and immediate fasciotomy is required to prevent irreversible damage [3]. Misdiagnosis represents an additional concern: because the clinical presentation often mimics DVT, patients may be unnecessarily anticoagulated or, more concerningly, a true DVT may be overlooked if symptoms are attributed solely to cyst rupture. Although improved diagnostic protocols, particularly routine duplex ultrasonography for calf swelling, have reduced such errors, vigilance remains essential [40-42].

Infection is an uncommon but recognized complication, particularly in the setting of septic arthritis or following aspiration procedures. In such cases, synovial fluid dissemination may lead to localized abscess formation within the calf, requiring antimicrobial therapy and possibly surgical drainage [1,9]. The existing literature on neuropathic complications, including tibial or peroneal nerve compression, is limited to isolated case reports, and there are no large-scale studies evaluating nerve outcomes [23,40].

Ruptured cysts can occur in the case of arthroscopic treatment for intra-articular lesions in the presence of a Baker’s cyst. The arthroscopic lavage water pump pressure can enlarge the cyst and represent a risk factor for rupture [43,44].

Conservative treatment remains the cornerstone of therapy and is appropriate for the vast majority of patients. Camanho et al. [12] recently reported a good result in 16 cases of ruptured Baker cyst treated conservatively with anti-inflammatory and physical therapy, with no recurrence at two-year follow-up.

Recent advances in imaging and minimally invasive techniques have improved the diagnostic and therapeutic landscape of popliteal cysts [41,42]. High-resolution ultrasonography and MRI have enhanced the ability to differentiate ruptured cysts from DVT and other causes of calf swelling, allowing more accurate evaluation of cyst anatomy, the presence of fluid dissection, and associated intra-articular pathology. These modalities have also contributed to the development of novel interventional approaches. Ultrasound-guided fenestration combined with corticosteroid injection has gained traction as a minimally invasive option that may offer improved outcomes over aspiration alone by promoting sustained cyst decompression [10]. Although promising, this technique requires further validation through larger prospective studies.

Surgical techniques have also evolved. Arthroscopy allows visualization of the cyst wall and the presence of cyst adherence. Arthroscopic management, which corrects intra-articular pathology and addresses the cyst-joint communication, has demonstrated favorable outcomes and reduced recurrence compared with historical open procedures [37-38]. Arthroscopic techniques can enlarge or unroof the communication, enabling the cyst to decompress effectively into the joint space [12]. Interest has also arisen in the use of sclerosing agents and biological therapies to modify synovial inflammation, though these remain experimental and are not widely adopted.

Fluid irrigation has to be performed with caution using low pressure or without the use of a pump, to avoid saline solution leakage through the ruptured cyst [26]. Surgical procedure against the cyst wall can be addressed with a shaver or radiofrequency.

Irismetov et al. [26] found a natural closure of the communications between the joint and the walls of the ruptured cyst in 10% of cases scheduled for surgical operation after more than a week since the rupture was diagnosed; in this case, the author recommended treating only the intra-articular underlying disease.

Despite these advances, significant gaps in the literature remain. Much of the current knowledge is derived from case reports, small case series, and level IV evidence, and few studies specifically focus on ruptured cysts as opposed to popliteal cysts in general [34-49]. Comparative trials evaluating conservative versus interventional therapies are lacking, and long-term outcome data are limited. Our study is a narrative review; given the heterogeneity of the included studies and the low levels of evidence, no statistical analysis was performed. Future research should include prospective studies assessing the effectiveness of interventional and arthroscopic techniques, as well as investigations into the biomechanical and inflammatory factors that predispose certain cysts to rupture. Improved education on the condition is also warranted, as misdiagnosis remains a clinically relevant issue.

## Conclusions

Rupture of a Baker’s cyst remains an under-recognized condition that can closely mimic DVT and other causes of acute calf swelling. This review demystifies its clinical presentation, clarifies the key diagnostic steps, and summarizes evidence-based management strategies to support accurate and timely decision-making. Early identification through appropriate imaging, particularly duplex ultrasonography and MRI, is essential to avoid misdiagnosis, unnecessary anticoagulation, and delays in recognizing true thrombotic events or rare complications such as compartment syndrome. Ruptured cysts usually resolve with conservative measures, reinforcing the importance of reassuring patients and focusing on symptom control while addressing the underlying intra-articular pathology. Image-guided interventions may be considered prior to surgical management, which is generally reserved for persistent or refractory cases.
